# Group II Metabotropic Glutamate Receptors: Role in Pain Mechanisms and Pain Modulation

**DOI:** 10.3389/fnmol.2018.00383

**Published:** 2018-10-09

**Authors:** Mariacristina Mazzitelli, Enza Palazzo, Sabatino Maione, Volker Neugebauer

**Affiliations:** ^1^Department of Pharmacology and Neuroscience, Texas Tech University Health Sciences Center, Lubbock, TX, United States; ^2^Section of Pharmacology L. Donatelli, Department of Experimental Medicine, University of Campania Luigi Vanvitelli, Naples, Italy; ^3^Center of Excellence for Translational Neuroscience and Therapeutics, Texas Tech University Health Sciences Center, Lubbock, TX, United States

**Keywords:** glutamate, pain, nociception, metabotropic glutamate receptor, mGluR, analgesia

## Abstract

Glutamate is the main excitatory neurotransmitter in the nervous system and plays a critical role in nociceptive processing and pain modulation. G-protein coupled metabotropic glutamate receptors (mGluRs) are widely expressed in the central and peripheral nervous system, and they mediate neuronal excitability and synaptic transmission. Eight different mGluR subtypes have been identified so far, and are classified into Groups I–III. Group II mGluR2 and mGluR3 couple negatively to adenylyl cyclase through Gi/Go proteins, are mainly expressed presynaptically, and typically inhibit the release of neurotransmitters, including glutamate and GABA. Group II mGluRs have consistently been linked to pain modulation; they are expressed in peripheral, spinal and supraspinal elements of pain-related neural processing. Pharmacological studies have shown anti-nociceptive/analgesic effects of group II mGluR agonists in preclinical models of acute and chronic pain, although much less is known about mechanisms and sites of action for mGluR2 and mGluR3 compared to other mGluRs. The availability of orthosteric and new selective allosteric modulators acting on mGluR2 and mGluR3 has provided valuable tools for elucidating (subtype) specific contributions of these receptors to the pathophysiological mechanisms of pain and other disorders and their potential as therapeutic targets. This review focuses on the important role of group II mGluRs in the neurobiology of pain mechanisms and behavioral modulation, and discusses evidence for their therapeutic potential in pain.

## Metabotropic Glutamate Receptors

The glutamatergic system provides excitatory neurotransmission throughout the central nervous system (CNS), and dysfunction of this system seems is correlated with several disorders such as schizophrenia, depression, and pain states. The interaction of glutamate with its ligand-gated cation channels (NMDA, AMPA, and kainate) mediates fast transmission and cell signaling while the activation of metabotropic glutamate receptors (mGluRs) stimulates intracellular pathways linked to various effector systems involved long-lasting modifications. First evidence for the ability of glutamate to stimulate the production of inositol phosphate (Sladeczek et al., 1985) formed the foundation for the cloning of the first mGluR (Masu et al., 1991). Numerous studies have greatly expanded the field. mGluRs belong to the G-protein coupled receptors (GPCRs) superfamily, and are classified in three groups based on sequence homology, signal transduction pathways and pharmacological agent selectivity (Schoepp et al., 1999; Neugebauer, 2007, 2015; Niswender and Conn, 2010; Nicoletti et al., 2011; Yin et al., 2014).

• Group I includes mGluR1 and mGluR5 coupled to G_q/11_ (Masu et al., 1991), and therefore their activation leads mainly to increased intracellular levels of inositol-1,4,5-trisphosphate (IP3) and diacylglycerol (DAG) via stimulation of phospholipase C_β_, although some evidence suggests the action on additional effector systems, such as mammalian target of rapamycin (mTOR) and the mitogen-activated protein kinase/extracellular receptor kinase (MAPK/ERK) pathways, key components of some form of synaptic plasticity (Hou and Klann, 2004; Page et al., 2006).
• Group II consists of mGluR2 and mGluR3, which are G_i/o_ coupled and promote the inhibition of adenylyl cyclase (Tanabe et al., 1992) and voltage-dependent calcium channels, as well as the activation of voltage-dependent potassium channels (Niswender and Conn, 2010; Nicoletti et al., 2011; Muguruza et al., 2016).
• Group III comprises mGluR4, mGluR7 and mGluR8, which similarly to group II mGluRs are negatively linked to G_i/o_ type proteins, and mGluR6, which is positively coupled to a cGMP phosphodiesterase (Neugebauer, 2008; Niswender and Conn, 2010).

The activity of those receptors can be modulated by pharmacological manipulations of the orthosteric binding sites with agonists, antagonists, or inverse agonists. The issue with the orthosteric ligands is the conservation of the binding site that makes it difficult to develop selective molecules for specific receptors. Another approach is the use of allosteric modulators that bind to specific sites of the receptors different from the orthosteric ones, and as a consequence they modulate the affinity of the receptor for its endogenous ligand. This binding is saturable such that no further effect is possible when all sites are occupied; better selectivity can be achieved because the allosteric binding sites present a lower evolutionary conservation compared to the orthosteric sites, and allosteric modulation can produce positive or negative effects based on the intrinsic activity of the compound (Conn et al., 2009; Montana and Gereau, 2011; Wood et al., 2011). Current research efforts are focused on the development of new positive and negative allosteric modulators (PAM and NAM) as potential therapeutic tools and strategies.

## Group II Metabotropic Glutamate Receptors

The group II mGluRs are found throughout the nervous system, including regions and circuits critically involved in nociceptive signaling and pain modulation as well as in emotional processing (Gu et al., 2008; Wright et al., 2013). They contribute to and modulate synaptic transmission and neuroplasticity, acting at the preterminal region away from the active zone of the synapse as autoreceptors on glutamatergic neurons or heteroreceptors on GABAergic neurons mediating a negative feedback signal (Nicoletti et al., 2011). As perisynaptic receptors, they are located on the pre-synaptic membrane distant from the synaptic cleft, where they can be activated by substantial synaptic glutamate release or astrocytic glutamate (Muguruza et al., 2016; Maksymetz et al., 2017). However, some evidence suggests that mGluR2 and mGluR3 are also expressed post-synaptically. Whereas mGluR2 seems to be present exclusively on neurons, mGluR3 is also found on glia cells (Muguruza et al., 2016). It is becoming clear now that group II mGluRs interact closely with other mGluRs, which has important functional implications. For example, mGluR2 forms a heterodimeric complex with mGluR4 that regulates the efficacies of mGluR2 and mGlu4 allosteric modulators (Yin et al., 2014), while mGluR3 and mGluR5 interact synergistically in the CNS through cross-talk of signaling pathways rather than by heterodimer interactions (Di Menna et al., 2018).

Therapeutic usefulness of compounds acting on group II mGluRs has been suggested for amyotrophic lateral sclerosis (Battaglia et al., 2015), schizophrenia, depression, anxiety (Patil et al., 2007; Fell et al., 2011; O’brien et al., 2014; Muguruza et al., 2016), drug addiction (Moussawi and Kalivas, 2010), Parkinson’s disease (Dickerson and Conn, 2012), and pain states (Neugebauer, 2007; Montana and Gereau, 2011; Neugebauer, 2013; Chiechio, 2016). The specific contribution of mGluR2 or mGluR3 subtypes has been challenging to determine because of the close similarity of the proteins that makes it difficult for pharmacological approaches to target these subtypes selectively. In order to better understand the role of individual mGluR subtypes, considerable effort has been dedicated to the development of more selective and CNS penetrant NAMs and PAMs, together with the development of mGluR knockout (KO) mice. While there is strong evidence for an over-activation of the glutamatergic system in pain states (Neugebauer, 2007; Zhou et al., 2011; Guida et al., 2015), and mGluRs in particular (Neugebauer, 2007; Montana and Gereau, 2011; Kolber, 2015), the role of the group II mGluRs and their subtypes in pain mechanisms and pain modulation is less well understood.

## Pharmacological Agents Targeting mGluR2/3

### Orthosteric Ligands

A number of molecules, classified according to their intrinsic activity and receptor selectivity, have been developed and tested in order to clarify the contribution of group II mGluRs to disease mechanisms and pathological conditions (**Table 1**). Most of the currently available compounds have effects also on other glutamate receptors (ionotropic or metabotropic) and do not differentiate between mGluR2 and mGluR3. For example, DCG-IV is a very potent and selective group II mGluR agonist but has also NMDA agonist effects (Zhou et al., 2011), while L-CCG-I is a potent but not very selective group II mGluR agonist and (2R,4R)-APDC (APDC) is highly selective group II mGluR2/3 agonist (Brabet et al., 1998; Schoepp et al., 1999). (1S,3S)-ACPD (ACPD) is the most selective mGluR2/3 agonist among the isomers of (±)-*cis*-ACPD (Hölscher et al., 1997). More selective and highly potent group II agonists, such as LY2934747, LY389795, LY354740, LY404039, LY379268 (also in the form of disodium salt), have been developed with good efficacy in animal models (Nicoletti et al., 2011, 2015; Caulder et al., 2014; Yin and Niswender, 2014; Maksymetz et al., 2017) including for pain states (Neugebauer et al., 2000; Simmons et al., 2002; Li and Neugebauer, 2006; Neugebauer, 2007; Chiechio and Nicoletti, 2012; Yin and Niswender, 2014; Johnson et al., 2017). SLx-3095-1 is the racemate (± isomers HCl salt) of the agonist LY379268 (– isomer) (Yamamoto et al., 2007). EGLU, LY341495 (also produced as disodium salt), and APICA are selective mGluR2/3 antagonists (Niswender and Conn, 2010; Yin and Niswender, 2014). Recently discovered LY3020371 seems to be even more selective for mGluR2/3 among all the mGluRs with potent effects in rat and human synaptosome preparations as well as in *in vivo* assays (Witkin et al., 2017).

**Table 1:** Drugsacting on mGluR2/3 tested in pain models.

	Compounds	Selectivity	Route of application	Pain model	Effect	Behaviors tests	Neural activity
Orthosteric agonists	DCG-IV	mGluR2/3, NMDA	i.th.	Spinal nerve ligation	Inhibition	Allodynia, mechanical hyperalgesia	
	L-CCG-I	Potent but not selective for mGluR2/3	Intra-PAG	Formalin	Inhibition	Paw-licking, lifting, shaking, flicking	
					Facilitation	Hot plate	
			Intra-spinal	Capsaicin	Inhibition		Spinothalamic tract – neuronal activity
	(1S,3S)-ACPD	mGluR2/3	i.th.	Carrageenan		Inhibition	Spinal dorsal horn – neuronal activity
	(2R,4R)-APDC	mGluR2/3	s.c.	PGE_2_-sensitization, carrageenan, formalin	Inhibition	Thermal hyperalgesia, allodynia	
			i.th.	Capsaicin	Inhibition	Allodynia	
				Capsaicin, Complete Freund’s adjuvant	No effect	Thermal hyperalgesia, allodynia	
			*Ex vivo* (skin nerve preparation and cultured DRG)	Capsaicin, PGE_2_-sensitization	Inhibition		Neuronal activity and calcium signals
	LY379268	mGluR2/3	i.p.	Formalin	Inhibition	Paw-licking	
				Spinal nerve ligation	Inhibition	Allodynia	
			*Ex vivo* (mPFC slice)	Kaolin-carrageenan-induced monoarthritis			mPFC – pyramidal output, excitatory transmission
	SLx-3095-1	mGluR2/3	*Ex vivo* (amygdala slice)	Formalin	Inhibition		Amygdala – neuronal activity, excitatory transmission
	LY354740	mGluR2/3	i.p.	Formalin	Inhibition	Paw-licking	
			Intra-amygdala and *ex vivo* (amygdala slice)	Kaolin-carrageenan-induced monoarthritis			Amygdala – neuronal activity, excitatory transmission
	LY389795	mGluR2/3	i.p.	Formalin	Inhibition	Paw- licking	
	LY2934747	mGluR2/3	Oral	Formalin, capsaicin, complete Freund’s adjuvant, plantar incision postsurgical, visceral (colorectal distension)	Inhibition	Allodynia, thermal and mechanical hyperalgesia	
			i.v.	Spinal nerve ligation	Inhibition	Allodynia	Spinal dorsal horn – neuronal activity
Orthosteric antagonists	EGLU	mGluR2/3	Intra-PAG	Formalin	No effect	Hotplate	
			Intra-reticular thalamic nucleus	Complete Freund’s adjuvant	Inhibition	Ankle-bend score	
			Intra-amygdala	Kaolin-carrageenan-induced monoarthritis	Facilitation		Amygdala – neuronal activity, excitatory transmission
	APICA	mGluR2/3	s.c.	PGE2-sensitization, carrageenan, capsaicin	Facilitation	Allodynia, paw-licking, flinching	
			*Ex vivo* (skin nerve preparation and cultured DRG)	Capsaicin			Nociceptive fiber activity; calcium signals
	LY341495	mGluR2/3	s.c.	PGE2-sensitization, carrageenan, capsaicin	Facilitation	Allodynia, paw-licking, flinching	
			i.th.	Complete Freund’s adjuvant	Inhibition	Allodynia	
					No effect	Thermal hyperalgesia	
			*Ex vivo* (skin nerve preparation and cultured DRG)	Capsaicin	Facilitation		Nociceptive fiber activity; calcium signals
			*Ex vivo* (mPFC slice)	Kaolin-carrageenan-induced monoarthritis			mPFC – pyramidal output, excitatory transmission


*See the sections “Pharmacological Manipulation of mGluR2/3 in Pain: Behavioral Studies” and “Pharmacological Manipulation of mGluR2/3 in Pain: Electrophysiological Studies” for references.*

### Allosteric Modulators

The development of selective NAMs and PAMs is now beginning to allow the targeting of mGluR2 and mGluR3 (Dhanya et al., 2010; Sheffler et al., 2011b; Bollinger et al., 2017). Only PAMs selectively binding to mGluR2, but not mGluR3, are available such as BINA, LY487379 hydrochloride, and CBIPES hydrochloride (Johnson et al., 2005; Dhanya et al., 2010; Sheffler et al., 2011b). These mGluR2 PAMs attenuated the ketamine-induced release of histamine in the medial prefrontal cortex (mPFC) (Fell et al., 2010) and decreased ketamine- or phencyclidine-induced hyperlocomotion (Sheffler et al., 2011b), suggesting antipsychotic activity of mGluR2. Selective NAMs are available for mGluR3 (LY2389575, VU0477950, and VU0650786) and more recently for mGluR2 (VU6001966) (Bollinger et al., 2017). LY2389575 established a key role of mGluR3 in neuroprotection against β-amyloid induced toxicity (Caraci et al., 2010; Sheffler et al., 2011a), suggesting that pharmacological activation of mGluR3 with PAMs may be a possible therapeutic strategy in Alzheimer’s disease. VU0477950 revealed a crucial role of mGluR3 in cognitive functions in mPFC-dependent fear extinction learning (Walker et al., 2015). VU0650786, an even more selective mGluR3 NAM, implicated the synergistic interaction of mGluR3 and mGluR5 in the generation of synaptic plasticity (long-term depression of excitatory transmission in cortical neurons) (Engers et al., 2015; Di Menna et al., 2018). Recently, VU6001966 emerged as a NAM for mGluR2 without any activity at the other mGluRs and with high CNS penetration (Bollinger et al., 2017; Di Menna et al., 2018).

## Pharmacological Manipulation of mGluR2/3 in Pain: Behavioral Studies

Pharmacological activation of group II mGluRs generally has antinociceptive effects in preclinical studies in rats and mice (Varney and Gereau, 2002; Neugebauer, 2007; Montana and Gereau, 2011; Chiechio, 2016). The activation of mGluR2/3 by systemically (intraperitoneally, i.p.) applied agonists (LY354740, LY379268, and LY389795) decreased nociceptive behavior in the late phase of the formalin test, a relatively acute pain model, in a dose-dependent manner, and the effect was reversed by a group II antagonist (LY341495) (Simmons et al., 2002). Systemic mGlu2/3 activation also decreased mechanical allodynia of neuropathic rats (spinal nerve ligation model, SNL), but had no effect in the tail flick test or the paw withdrawal latency test (acute thermal pain models) (Simmons et al., 2002). Importantly, the antinociceptive effect of systemically (i.p.) applied LY379268 in the formalin pain model was lost in mGluR2, but not mGluR3, knock-out mice (Zammataro et al., 2011), suggesting an important role of mGluR2. Recently, oral application of a prodrug (LY2969822) for the selective mGluR2/3 agonist LY2934747 has been reported to have antinociceptive effects in various preclinical models of inflammatory (formalin, capsaicin, complete Freund’s adjuvant [CFA]), postsurgical (plantar incision), visceral (colorectal distension), and neuropathic (SNL) pain (Johnson et al., 2017).

### Peripheral

Subcutaneous injection of a group II mGluR agonist (APDC) into the plantar surface of the hindpaw did not change baseline mechanical and thermal sensitivity but blocked prostaglandin E_2_ (PGE_2_)-induced thermal hyperalgesia, PGE_2_- or carrageenan-induced mechanical allodynia, and nociceptive responses in both phases of the formalin test; these antinociceptive effects were inhibited by co-application of a group II mGluR antagonist (LY341495) (Yang and Gereau, 2002, 2003; Yamamoto et al., 2007). Interestingly, group II antagonists (LY341495 and APICA) alone prolonged the mechanical allodynia in the PGE2 and carrageenan models and increased nociceptive behaviors in the capsaicin model, supporting the hypothesis of endogenous mGluR2/3 activation in inflammatory pain conditions (Yang and Gereau, 2002, 2003; Carlton et al., 2011). The data are consistent with antinociceptive effects of peripheral group II mGluR activation, although there may be species differences (Sheahan et al., 2018; see the sections “Peripheral” and “Clinical Trials and Potential Clinical Uses”).

### Spinal

Intrathecal (i.th.) administration of a selective group II mGluR agonist (APDC) in the absence of tissue damage had no effect on mechanical thresholds (von Frey test) and thermal paw withdrawal latencies but inhibited capsaicin-induced mechanical allodynia without affecting thermal hyperalgesia (Soliman et al., 2005). However, intrathecal APDC had no effect in an inflammatory pain model induced by subcutaneous (s.c.) CFA injection into the hindpaw (Zhang et al., 2009). In neuropathic rats (SNL model) intrathecal application of a group II mGluR agonist (DCG-IV) decreased mechanical allodynia (von Frey test) and mechanical hyperalgesia (paw withdrawal threshold to noxious pressure stimuli) in a dose-dependent way, and these antinociceptive effects were blocked by a group II mGluR antagonist (EGLU) (Zhou et al., 2011). Interestingly, intrathecal application of DCG-IV had a pronociceptive effect in sham rats, which was reversed by an NMDA receptor antagonist (AP-5), suggesting that this effect was mediated by the activation of NMDA receptors (Zhou et al., 2011). Intrathecal application of a group II mGluR antagonist (LY341495) ameliorated mechanical allodynia, but not thermal hyperalgesia, in the CFA-induced inflammatory pain model; the antinociceptive effect was potentiated by a glial cells inhibitor (fluorocitric acid) (Zhang et al., 2009). These mixed and somewhat inconsistent effects of group II mGluR compounds may be due to a lack of subtype-specificity and/or reflect rather complex functions of group II mGluRs in spinal nociceptive processing.

### Brainstem

In the periaqueductal gray (PAG), activation of group II mGluRs had pronociceptive effects under normal conditions but antinociceptive effects in an acute pain model. Microinjection of a group II agonist (L-CCG-I) into the dorsolateral PAG dose-dependently inhibited the nociceptive responses (lifting, licking, shaking and flicking the injected paw) in the late phase of the formalin test (acute pain model) (Maione et al., 2000), but had a dose-dependent pronociceptive effect in the hot plate test, decreasing the latency of the nociceptive responses (licking the paw; jumping) (Maione et al., 1998). Both effects were counteracted by the intra-PAG administration of a group II mGluR antagonist (EGLU) (Maione et al., 1998, 2000). EGLU alone had no effect in the hotplate test (Maione et al., 1998). Together with microdialysis data showing that L-CCG-I increased serotonin release in the PAG in a GABA_A_ receptor dependent way (Maione et al., 1998), these results were interpreted to suggest that group II mGluRs in the PAG promote an antinociceptive effect mainly by decreasing GABA release to potentiate the activity of the descending antinociceptive pathway following persistent noxious stimulation (Maione et al., 2000).

### Brain

In the thalamus group II mGluRs mediate the presynaptic inhibition of GABAergic inhibitory transmission from the reticular thalamic nucleus to the somatosensory ventrobasal thalamus (VB) (Salt and Turner, 1998) to facilitate sensory processing through an action on mGluR2 (Copeland et al., 2012) possibly on astrocytes (Copeland et al., 2017). Stereotaxic administration of a group II mGluR antagonist (EGLU) into the reticular thalamic nucleus, but not other thalamic nuclei, had an antinociceptive effect in an arthritis pain model (complete Freund’s adjuvant-induced monoarthritis in the ankle joint), reducing the ankle-bend test scores, possibly through a mechanism that involves blocking the disinhibition of somatosensory thalamic relay neurons (Neto and Castro-Lopes, 2000).

### Subtype Selective Interventions

Behavioral effects of negative and positive allosteric modulators for mGluR2 and mGluR3 remain to be determined in pain conditions, but the contribution of individual subtypes is being addressed using alternative approaches.

*N-acetylcysteine (NAC)* has been used to probe mGluR2 function in pain models. NAC promotes the activity of the L-cystine/L-glutamate membrane exchanger (Sxc-), a crucial antiporter for the release of glutamate from astrocytes for the endogenous activation of perisynaptic mGluR2/3 (Kalivas, 2009). NAc may therefore be used to increase endogenous activation of these receptors (Chiechio and Nicoletti, 2012). Systemic (i.p.) application of NAC inhibited nocifensive behaviors in the tail flick test (Truini et al., 2015) and in the second phase of the formalin test (Bernabucci et al., 2012), and decreased mechanical hypersensitivity in an inflammatory pain model (subcutaneous CFA in the hindpaw) and in a neuropathic pain model (chronic constriction injury, CCI) (Bernabucci et al., 2012). The effects of NAC were blocked by an mGluR2/3 antagonist (LY341495) (Bernabucci et al., 2012). The antinociceptive effect of NAC in the formalin pain model was lost in mGluR2, but not mGluR3, knockout mice (Bernabucci et al., 2012), which points to an action on mGluR2.

Drug-induced potentiation of the transcription of GRM2, the gene encoding for mGluR2, has been used to assess antinociceptive effects of increased expression of mGluR2 in dorsal root ganglia and spinal dorsal horn (see Chiechio and Nicoletti, 2012). Indeed, *epigenetic drugs* such as LAC (Chiechio et al., 2002) and HDAC inhibitors (Chiechio et al., 2009) showed antinociceptive effects in different pain models. Systemic (s.c.) application of LAC decreased mechanical and thermal hypersensitivity in a neuropathic pain model (CCI) through increased expression of mGluR2 but not mGluR3 (Chiechio et al., 2002). Systemic (s.c.) application of HDAC inhibitors reduced the nociceptive response in the second phase of the formalin test by up-regulation of mGluR2 expression (Chiechio et al., 2009). Spinal (i.th.) administration of HDAC inhibitors attenuated the pronociceptive effect of estrogen on visceral sensitivity (increased visceromotor response to colorectal distension) and increased mGluR2 but not mGluR3 expression (Cao et al., 2015), which is consistent with a predominant action on mGluR2.

For the study of mGluR3 function, the neuropeptide *N-acetylaspartylglutamate (NAAG)* has been tested as a preferential activator of mGluR3 (Neale et al., 2000; Neale, 2011). Consistent with its wide distribution throughout the nervous system, local peripheral (s.c.) application of NAAG inhibited mechanical allodynia in the carrageenan-induced hindpaw inflammatory pain model (Yamamoto et al., 2007) and intracerebroventricular (i.c.v.) administration was antinociceptive in both phases of the formalin pain test (Yamamoto et al., 2008). Another strategy to target mGluR3 is to increase NAAG levels with NAAG peptidase inhibitors such as ZJ-11, ZJ-17 and ZJ-43, ZJ-45 or 2-PMPA, to block NAAG degradation (Neale et al., 2005). NAAG peptidase inhibitors administered systemically or peripherally or locally into CNS regions had antinociceptive effects in models of inflammatory and neuropathic pain. Systemic (intravenous, i.v., or i.p.) application decreased nociceptive behaviors (flinching) in both phases of the formalin pain test (Yamamoto et al., 2004; Nonaka et al., 2017) and had antiallodynic effects in a neuropathic pain model (partial sciatic nerve ligation) without affecting baseline mechanical and thermal sensitivity in the von Frey and hot plate tests, respectively (Yamamoto et al., 2004). Peripheral (s.c.) injection also decreased both phases of the formalin pain test and had anti-allodynic effects in the carrageenan pain model (Yamamoto et al., 2007). Spinal (i.th.) application inhibited nocifensive responses (flinching) in both phases of the formalin pain test and mechanical allodynia in the partial sciatic nerve ligation model, but had no effect in the von Frey and hot plate tests (Yamamoto et al., 2004). Microinjections into PAG or rostral ventromedial medulla (RVM) inhibited nociceptive behaviors (flinching) in both phases of the formalin pain test but had no effect in the hot plate test (Yamada et al., 2012). Injections into the locus coeruleus had similar antinociceptive effects in the formalin test (Nonaka et al., 2017). Intracerebroventricular administration also reduced nociceptive behaviors in both phases of the formalin pain test response (Yamamoto et al., 2008). Antinociceptive effects were blocked with a group II mGluR antagonist (LY341495) where tested in these studies. While there has been some controversy regarding the selective activation of mGluR3 with NAAG and peptidase inhibitors (for Discussion, see Neale, 2011) studies from mGluR2 and mGluR3 knockout mice provide strong evidence for mGluR3 mediated effects (Olszewski et al., 2017).

## Pharmacological Manipulation of mGluR2/3 in Pain: Electrophysiological Studies

Effects of group II mGluR agonists or antagonists on pain-related neuronal activity were studied in primary sensory neurons, spinal dorsal horn, and a few brain regions (amygdala and mPFC). Drugs were typically administered locally and there is surprisingly little information available for neuronal effects of systemic drug application and their site(s) of action. To the best of our knowledge, only a recent study showed inhibitory effects of a systemically (i.v.) applied mGluR2/3 agonist (LY2934747) on extracellularly recorded background activity and electrically evoked (C-fiber stimulation) wind-up discharges of dorsal horn neurons in neuropathic rats (SNL model) (Johnson et al., 2017).

### Peripheral

A group II agonist (APDC) inhibited extracellularly recorded activity (action potentials) of nociceptive fibers evoked by capsaicin or by an inflammatory soup, and blocked the inflammatory soup- or forskolin-induced sensitization of heat responses in an *in vitro* rat skin-nerve preparation (Du et al., 2008). APDC had no effect on baseline heat or mechanical thresholds or discharges (Du et al., 2008; Carlton et al., 2011). APDC reversed the PGE_2_-induced hyperexcitability of cultured mouse and human primary sensory (dorsal root ganglia, DRG) neurons (Davidson et al., 2016). Interestingly, APDC blocked the PGE_2_-induced sensitization of capsaicin responses (calcium influx) in cultured mouse, but not human, DRG neurons (Yang and Gereau, 2002; Sheahan et al., 2018) through a mechanism that involved Gi dependent inhibition of adenylyl cyclase (Yang and Gereau, 2002).

Group II mGluR antagonists (LY341495 or APICA) enhanced the capsaicin-induced action potentials of nociceptive fibers in the skin nerve preparation or calcium signals in DRG neurons, but had no effect alone, suggesting that group II mGluRs act endogenously to reverse hypersensitivity (Carlton et al., 2011). In presence of excess extracellular glutamate in the skin nerve preparation, the blockade of the group II mGluRs also increased activity and heat responses of nociceptive fibers, which was interpreted to suggest that GluR2/3 activation by the exogenous glutamate decreased nociceptor activity and this activity-dependent autoinhibition of nociceptive signal transmission to the CNS would modulate pain sensitivity (Carlton et al., 2011).

### Spinal

Intrathecal administration of a group II agonist, ACPD, inhibited electrically evoked C-fiber responses of dorsal horn neurons recorded extracellularly in anesthetized rats with a carrageenan-induced hindpaw inflammation (3 h postinduction), but had mixed (excitatory or inhibitory) effects in normal animals (Stanfa and Dickenson, 1998). Administration of group II mGluR agonists (LY379268 and L-CCG-I) into the spinal dorsal horn by microdialysis decreased the central sensitization of primate (*Macaca fascicularis*) spinothalamic tract cells induced by intradermal capsaicin (30 min postinduction), but had no effect on the responses of non-sensitized neurons to innocuous and noxious cutaneous mechanical stimuli (Neugebauer et al., 2000). Information about the role of spinal group II mGluRs and their subtypes nociception and pain models is surprisingly thin, and given the mixed results of behavioral studies (see the section “Spinal”) the spinal cord may not be the main target of their overall beneficial effects related to pain.

### Brain

Actions of group II mGluR compounds have been studied in the amygdala, a key player in emotions, emotional aspects of pain and pain modulation (Neugebauer et al., 2004; Thompson and Neugebauer, 2017), and in the mPFC, a center for executive functions and behavioral control related to negative emotions and pain (Neugebauer et al., 2009; Ong et al., 2018). In anesthetized rats, stereotaxic administration (microdialysis) of a group II mGluR agonist (LY354740) into the central nucleus of the amygdala (CeA) targeting its laterocapsular division (CeLC), which is also referred to as the “nociceptive amygdala,” decreased the responses of CeLC neurons to innocuous and noxious mechanical stimuli under normal conditions, but became more potent in an arthritis pain model (kaolin-carrageenan-induced monoarthritis in the knee) (Li and Neugebauer, 2006). The agonist effects were blocked by co-administration of a group II antagonist (EGLU), which by itself had no effect under normal conditions but increased the evoked responses to noxious stimulation of the arthritic knee in the pain model (Li and Neugebauer, 2006). This would be consistent with endogenous activation and gain of function of mGluR2/3 in the amygdala in a pain condition. Patch-clamp recordings of CeLC neurons in rat brain slices showed that a group II mGluR agonist (LY354740) inhibited excitatory synaptic inputs (EPSCs) from the parabrachial area, which provide nociceptive information to the amygdala (Neugebauer et al., 2004; Thompson and Neugebauer, 2017), under normal conditions, but became more potent in the arthritis pain condition (Han et al., 2006; Kiritoshi and Neugebauer, 2015). LY354740 decrease frequency, but not amplitude, of miniature EPSCs in the presence of TTX, suggesting a presynaptic site of action on the glutamatergic terminals (Han et al., 2006; Kiritoshi and Neugebauer, 2015). EGLU blocked the agonist effect, but had no effect on its own, which is similar to the lack of significant effects of another group II mGluR antagonist (LY341495) at the presumed parabrachial (PB)-CeLC synapse in mouse brain slices (Adedoyin et al., 2010), suggesting that the endogenous activation observed in the *in vivo* condition (see above) may be lost in the reduced brain slice preparation (Han et al., 2006; Kiritoshi and Neugebauer, 2015).

In the infralimbic mPFC, rat brain slice physiology experiments found that group II mGluRs decreased the output of principal layer V pyramidal cells as the result of an inhibitory action on glutamatergic synapses under normal conditions and in an arthritis pain model (kaolin-carrageenan-induced monoarthritis in the knee), and that this system was tonically active under both conditions (Kiritoshi and Neugebauer, 2015; Thompson and Neugebauer, 2017). Specifically, a selective group II mGluR agonist (LY379268) decreased synaptically evoked spiking of pyramidal cells in brain slices from normal and arthritic rats by inhibiting direct excitatory inputs (EPSCs) as well as glutamate-driven feedforward inhibitory transmission (IPSCs) (Kiritoshi and Neugebauer, 2015; Thompson and Neugebauer, 2017). Abnormally enhanced synaptic inhibition of mPFC output in pain conditions has been linked to cognitive dysfunction (Ji et al., 2010; Kiritoshi et al., 2016) and loss of cortical control of amygdala function (Ji and Neugebauer, 2014; Kiritoshi and Neugebauer, 2018). Effects of LY379268 on EPSCs preceded those on IPSCs, resulting in a net inhibitory effect on pyramidal output. Spontaneous and miniature (in TTX) analyses of EPSCs and IPSCs showed that LY379268 acted presynaptically on glutamatergic, but not GABAergic, terminals. The effects of LY379268 were blocked by a selective group II mGluR antagonist (LY341495) that by itself increased synaptically evoked spiking of pyramidal cells under normal conditions and in the pain model (Kiritoshi and Neugebauer, 2015; Thompson and Neugebauer, 2017), suggesting endogenous activation of mGluR2/3. It has been speculated that failure to release this inhibitory tone to enhance mPFC output could be a mechanism of pain persistence due to a lack of cortical control (Kiritoshi and Neugebauer, 2015; Thompson and Neugebauer, 2017).

### Subtype Selective Interventions

In mouse amygdala brain slices, exogenous NAAG and a NAAG peptidase inhibitor (ZJ-43) (see the section “Subtype Selective Interventions”) have been tested in order to elucidate the specific contribution of mGluR3. Under normal conditions, NAAG and ZJ-43 inhibited excitatory transmission (EPSCs) at the presumed PB-CeLC synapse (see the section “Brain”) similarly to an mGluR2/3 agonist (SLx-3095-1) (Adedoyin et al., 2010), which is consistent with an mGluR3 effect. The effect of ZJ-43 was blocked by a group II mGluR antagonist (LY341495). In the formalin pain model (brain slices taken 24 h postinduction), ZJ-43 was much less efficacious than SLx-3095-1 in inhibiting EPSCs, suggesting a decreased release of NAAG or an increased contribution of mGluR2 rather than mGluR3 in the pain condition (Adedoyin et al., 2010).

## Clinical Trials and Potential Clinical Uses

Pain conditions affect millions of people, and pain management can be challenging and often is insufficient with currently available tools. Based on several lines of evidence from preclinical studies, drugs acting on mGluR2/3 may be useful for pain relief, but so far have not advanced to clinical trials as analgesic candidates, perhaps because of concerns about the translation from animal models to the human condition (Davidson et al., 2016). However, despite concerns for example about the development of tolerance in some rodent studies, there is no evidence for a loss of efficacy on repeat dosing of group II mGluR agonists in humans (Johnson et al., 2017). In fact, significant anxiolytic efficacy of an oral prodrug (LY544344) of an mGluR2/3 agonist (LY354740) was observed in patients with generalized anxiety (Dunayevich et al., 2008). An oral prodrug (LY2140023, pomaglumetad methionil) of an mGluR2/3 agonist (LY404039) had significant antispychotic efficacy in schizophrenia patients (Patil et al., 2007) or in subgroups of schizophrenia patients (Kinon et al., 2015; Nisenbaum et al., 2016). In these studies, drug effects were maintained or enhanced following several weeks dosing (Johnson et al., 2017). It should be noted that NAC, which has been linked to the endogenous activation of mGluR2/3 (see the section “Subtype Selective Interventions”), given orally to healthy human subjects, decreased thermal pain ratings to laser stimuli and amplitudes of laser-evoked brain potentials without affecting thermal pain thresholds (Truini et al., 2015).

## Conclusion

Preclinical studies suggest that group II mGluRs play a significant role in the modulation of nociception and pain conditions. There is some evidence to suggest distinct roles of mGluR2 and mGluR3 subtypes in different neural circuits and regions, but this remains to be determined more thoroughly with the availability of more selective compounds such as allosteric modulators. These new tools are also useful for the analysis of the pathophysiological mechanisms of pain conditions. Effectiveness of mGluR2/3 compounds in clinical studies on conditions other than pain may support their therapeutic potential for the management of pain (Johnson et al., 2017).

## Author Contributions

MM collected information and provided first draft of manuscript. EP and SM provided valuable input to the manuscript. VN conceived the project and finalized the manuscript.

## Conflict of Interest Statement

The authors declare that the research was conducted in the absence of any commercial or financial relationships that could be construed as a potential conflict of interest.

## Abbreviations

ACPD: (1S,3S)-1-aminocyclopentane-1,3-dicarboxylic acid
AP-5: (-)-2-amino-5-phosphonopentanoic acid
APDC: (2R,4R)-4-aminopyrrolidine-2,4-dicarboxylate
APICA: (RS)-1-amino-5-phosphonoindan-1-carboxylic acid
BINA: 4-[3-[(2-cyclopentyl-6,7-dimethyl-1-oxo-2,3-dihydroinden-5-yl)oxymethyl]phenyl]benzoic acid
CBiPES hydrochloride: *N*-(4′-cyano-[1,1′-biphenyl]-3-yl-*N*-(3-pyridinylmethyl)-ethanesulfonamide hydrochloride
CBIPES: *N*-(4′-cyano-biphenyl-3-yl)-*N*-(3-pyridinylmethyl)-ethanesulfonamide hydrochloride
DGC-IV: (2S,1′R,2′R,3′R)-2-(2,3-dicarboxycyclopropyl)glycine
HDAC: histone deacetylase
L-CCG-I: (2S, 1S, 2S)-2-(carboxycyclopropyl)glycine
LAC: L-acetylcarnitine
LY341495: (1S,2S)-2-[(2S)-2-amino-3-(2,6-dioxo-3H-purin-9-yl)-1-hydroxy-1-oxopropan-2-yl]cyclopropane-1-carboxylic acid
LY379268: (-)-2-oxa-4-aminobicyclo[3.1.0]hexane-4,6-dicarboxylate
LY354740: 2-aminobicyclo[3.1.0]hexane 2,6-dicarboxylate
LY487379: 2,2,2-trifluoro-*N*-[4-(2-methoxyphenoxy) phenyl]-*N*-(3-pyridinylmethyl)ethanesulfonamide
NAAG: *N*-acetylaspartylglutamate
NAC: *N*-acetylcysteine
2-PMPA: 2-(phosphonomethyl)pentanedioic acid
SLx-3095-1: (+/-)-2-oxa-4-aminobicyclo[3.1.0]hexane-4,6-dicarboxylate
TTX: tetrodotoxin
ZJ-11: (S)-2-[3-[(S)-1-carboxy-3-(methylsulphanyl)propyl]ureido]pentanedioic acid
ZJ-17: (S)-2-[3-[(S)-1-carboxy-2-(4-hydroxyphenyl)ethyl]ureido]pentanedioic acid
ZJ-43: (S)-2-[3-[(S)-1-carboxy-3-methylbutyl]ureido]pentanedioic acid
